# Sarcopenia in amyotrophic lateral sclerosis: a key predictor of respiratory dysfunction and disease progression

**DOI:** 10.3389/fnut.2026.1713253

**Published:** 2026-03-02

**Authors:** María Teresa Zarco-Martín, María Carmen Andreo-López, Miriam Soraya Yagui-Beltrán, María Luisa Fernández-Soto

**Affiliations:** 1 Endocrinology and Nutrition Unit, San Cecilio University Hospital, Granada, Spain; 2 Foundation for Health Research in Eastern Andalusia – Alejandro Otero (FIBAO), Granada, Spain; 3 Granada Biosanitary Research Institute (Ibs. Granada), Granada, Spain; 4 Department of Medicine, University of Granada, Granada, Spain

**Keywords:** amyotrophic lateral sclerosis, disease severity, muscle mass, respiratory decline, sarcopenia

## Abstract

**Background:**

Amyotrophic Lateral Sclerosis (ALS) is a neurodegenerative disease characterized by progressive muscle weakness and respiratory decline. Sarcopenia remains underexplored in terms of prevalence and their relationship with disease progression. We aimed to determine the prevalence of sarcopenia in ALS patients, assess the predictive value of morphofunctional assessment tools for sarcopenia, and explore their relationship with respiratory function and disease progression.

**Methods:**

A cross-sectional study was conducted with 40 ALS patients at the ALS Multidisciplinary Unit, San Cecilio University Hospital in Granada. Sarcopenia was defined based on the European Working Group of Sarcopenia in Older People 2(EWGSOP2) and malnutrition was diagnosed using GLIM criteria. Morphofunctional status was assessed using: Phase Angle (PA) and body composition by Bioelectrical Impedance Vector Analysis, muscle strength through Handgrip Strength (HGS). Respiratory function was evaluated using Forced Vital Capacity (FVC). Associations between sarcopenia, body composition, respiratory function, and disease severity were analyzed using logistic regression models. Receiver operating characteristic analyses were performed to identify optimal predictive cut-off values.

**Results:**

Sarcopenia was identified in 25% of ALS patients. Compared with non-sarcopenic individuals, sarcopenic patients exhibited significantly lower muscle mass indices, PA, and HGS, along with higher extracellular water percentage (%ECW). Malnutrition was more frequent in sarcopenia group (90% vs. 25%, *p* < 0.001). Respiratory impairment was more pronounced in sarcopenic patients, with reduced FVC and elevated pCO₂ (*p* = 0.02), and a greater need for non-invasive mechanical ventilation (NIMV) (70% vs. 10%, *p* = 0.001). VC correlated positively with body cell mass index (BCMI) (*r* = 0.450), skeletal muscle mass index (SMI) (*r* = 0.413), and ALSFRS-R score (*r* = 0.731; all *p* < 0.05). Lower PA, BCMI, and ALSFRS-R scores, together with higher %ECW and partial pressure of carbon dioxide (pCO₂), predicted sarcopenia risk. Reduced BCMI, HGS, Short Physical Performance Battery (SPPB) and sarcopenia were associated with the need of NIMV. BCMI (cut-off:8.05 kg/m^2^; AUC:0.889) and ALSFRS-R (cut-off:33 points; AUC:0.884) were the most accurate predictors of sarcopenia and ventilatory support, respectively.

**Conclusion:**

This study is the first to assess sarcopenia prevalence in ALS patients using standardized diagnostic criteria. The findings highlight the relationship between sarcopenia, malnutrition, and respiratory decline. PA, BCMI, and respiratory parameters emerge as potential tools for sarcopenia and NIMV risk stratification.

## 1 Introduction

Amyotrophic Lateral Sclerosis (ALS) is a progressive neurodegenerative disease characterized by the selective loss of motor neurons in the brain and spinal cord, leading to widespread muscle atrophy, functional decline, respiratory failure and death within 3–5 years (1). While motor neuron denervation is the primary driver of muscle loss, growing evidence suggests that nutritional deficits, metabolic dysregulation, and systemic alterations further contribute to muscle wasting and disease progression (2, 3).

ALS presents with two main clinical phenotypes: bulbar-onset and limb-onset disease. Bulbar-onset ALS involves early swallowing impairment, accounts for 20%–35% of cases and is associated with faster progression and shorter survival. Limb-onset ALS begins with distal weakness in the limbs and progresses more gradually (4). Consequently, bulbar-onset ALS is associated with earlier nutritional intervention and an earlier and more frequent need for oral and/or gastrostomy tube enteral feeding. Limb-onset ALS tends to have a slower progression to nutritional deterioration (5). Both bulbar- and limb-onset ALS are associated with a high risk of sarcopenia, but the mechanisms and timing differ. In bulbar-onset ALS, early dysphagia and malnutrition accelerate muscle wasting and loss of muscle function, leading to rapid development of sarcopenia (6). Limb-onset ALS also results in progressive muscle and fat wasting, with body composition changes (decreased fat-free mass and fat mass) correlating with disease progression and nutritional status (7).

Sarcopenia emerges as a relevant clinical feature of ALS. Defined by the progressive loss of muscle strength (dynapenia), mass (myopenia) and function, it results from multifactorial mechanisms such as oxidative stress, mitochondrial dysfunction, and chronic inflammation (8). In ALS, sarcopenia may further compromise functional independence, nutritional status, and respiratory function, thereby increasing disease burden and reducing survival (6). Despite its potential impact, sarcopenia remains underrecognized in ALS and its contribution to ventilatory decline and disease progression is not yet fully understood.

In contrast, malnutrition has been extensively studied as a prognostic factor in ALS, largely due to its impact on muscle catabolism and energy imbalance (9). Notably, malnutrition and sarcopenia share overlapping characteristics, particularly reduced muscle mass (6), and might act synergistically to exacerbate functional decline.

Respiratory muscle dysfunction is a hallmark of ALS progression and diaphragmatic weakness is the primary driver of ventilatory failure (10). Since the diaphragm is a skeletal muscle, it is highly dependent on the integrity of motor neurons and the preservation of muscle mass. Therefore, sarcopenia in people with ALS contributes to diaphragmatic dysfunction through atrophy, reduced contractility and altered muscle signaling pathways. As the diaphragm weakens, patients develop hypoventilation, especially during sleep when accessory muscles are inactive and the diaphragm becomes the primary inspiratory muscle. This leads to nocturnal hypoxemia, hypercapnia, and sleep fragmentation, which manifest clinically as morning headaches, daytime fatigue, and dyspnea (11, 12). The cumulative impact of these symptoms includes progressive respiratory disability, reduced exercise tolerance and impaired activities of daily living, all of which are major determinants of diminished quality of life in ALS (13, 14). Early recognition and intervention targeting both nutritional status and respiratory muscle function are essential to mitigate these effects. Importantly, structural changes secondary to diaphragmatic atrophy are not reliably predicted by routine respiratory measures, supporting the need for direct assessment of muscle mass and function (15).

Morphofunctional assessment, which integrates body composition, muscle strength and physical performance, offers a comprehensive framework for evaluating muscle health status (8). Traditional measures such as weight or body mass index (BMI), although established prognostic markers in ALS (16), often fail to detect early muscle depletion and functional loss. In contrast, bioelectrical impedance vector analysis (BIVA) has been validated in the ALS population, and phase angle has been associated with prognosis and disease severity in this condition (17). Hand-grip strength, while widely used to assess muscle function in neuromuscular disorders (18), remains less well studied in ALS. When combined with respiratory function assessments, these tools may offer a more accurate characterization of muscle quality and functional reserve, supporting improved risk stratification and more individualized clinical management.

This study aimed to explore sarcopenia prevalence and to assess differences in body composition, functional status, respiratory function, and disease severity according to sarcopenia diagnosis in ALS patients using standardized diagnostic criteria. We also sought to identify key morphofunctional predictors of sarcopenia and examine their relationship with ventilatory decline and disease progression.

## 2 Methods

### 2.1 Study design and population

A cross-sectional observational study including 40 adult patients diagnosed with ALS who attended a nutrition consultation in the multidisciplinary team of ALS (UMELA) at San Cecilio University Hospital in Granada was conducted between March 2023 and January 2025. People with neurodegenerative diseases other than ALS were excluded. All adults with ALS were diagnosed based on El Escorial criteria (19).

The sample was determined based on feasibility. Given the low prevalence of ALS and the extensive morphofunctional and respiratory assessments performed, the sample size was constrained by practical considerations.

The study was approved by the Ethics Committee of Granada (Spain) following the principles of the World Medical Association Declaration of Helsinki. The project was reviewed and approved by the Research Ethics Committee of Granada on February 25, 2022 (ID: 1770-N-21). All participants provided oral and written informed consent.

### 2.2 Clinical variables

Clinical variables included evolution of the disease since ALS diagnosis (months), type of symptomatology onset (bulbar/spinal), malnutrition diagnosis and respiratory variables.

Malnutrition was diagnosed according to the Global Leadership Initiative on Malnutrition (GLIM) criteria. This is a standardized framework for diagnosing malnutrition based on the presence of at least one phenotypic criterion (weight loss, low BMI, or reduced muscle mass) and at least one etiologic criterion (reduced dietary intake and/or disease burden/inflammation). To assess muscle mass, we used appendicular skeletal mass index (ASMI) calculated using a predictive equation. To classify normality, the cut-off points used were males > 7 kg/m^2^ and females > 6 kg/m^2^ (20).

Respiratory data were collected from clinical history including forced vital capacity (FVC in %), peak cough flow (PCF in L), CO_2_ pressure (pCO_2_ in mmHg), use of cough assist (CA). Spirometry was performed using Vyntus™ SPIRO (Jaeger Medical), which includes internal quality control parameters to ensure accurate measurements. The maneuvers were conducted according to ATS/ERS guidelines, and only technically acceptable and reproducible attempts were included (21). Direct measurements of respiratory muscle strength (maximal inspiratory and expiratory pressures (MIP, MEP), and sniff nasal inspiratory pressure (SNIP)) were not consistently performed in all patients during the study period. Information regarding noninvasive mechanical ventilation (NIMV) was collected from clinical records. Patients were ventilated using portable ventilators. These devices allow manual adjustment of tidal volume (Vt) in milliliters, but do not automatically set Vt based on ideal or actual body weight. Settings were individualized by the treating clinicians according to patient condition and tolerance.

The ALSFRS-R (Revised Amyotrophic Lateral Sclerosis Functional Rating Scale) score was used to assess ALS severity. The scale comprises 12 items designed to measure functional abilities in daily activities across the disease course. The total score ranges from 0 to 48, in which higher scores indicate better physical function (22).

### 2.3 Morphofunctional assessment and disease-related sarcopenia diagnosis

Morphofunctional assessment encompasses a comprehensive set of techniques designed to evaluate both body composition and functionality, integrating quantitative and qualitative analyses to provide a deeper understanding of muscular and metabolic status (23).

Sarcopenia was defined as low muscle strength (dynapenia) and low muscle mass (myopenia) according to the European Working Group on Sarcopenia in Older People 2 (EWGSOP2). Dynapenia criteria is defined in Section 2.3.1. Myopenia was assessed by ASMI (men < 7 kg/m^2^, women < 5.5 kg/m^2^) (8). The SPPB functional test was used to assess severity, defined in section 2.3.2.

#### 2.3.1 Muscle strength

Muscle strength was determined using a calibrated handgrip dynamometer (Jamar, Asimow Engineering Co., Los Angeles, CA, USA). Assessments were conducted on the dominant hand, performing three trials with brief rest intervals. The highest value obtained was recorded as the individual’s handgrip strength (HGS). Cut-off values were established according to the EWGSOP2 recommendations, considering <27 kg in men and <16 kg in women as indicative of low muscle strength (8).

#### 2.3.2 Physical performance

Physical performance was analyzed using the Short Physical Performance Battery (SPPB), which includes 3 domains: balance, walking speed, and getting up from and sitting down on a chair 5 times (24).

Balance performance was evaluated through three progressively challenging stances: feet together, semi-tandem, and full tandem, each maintained for 10 s. The sequence was administered in hierarchical order. Gait speed was assessed over a 4-meter walk at the participant’s usual pace, with the completion time recorded in seconds. The chair stand test required participants to rise from and sit back on a chair five times as quickly as possible, and the total duration was documented.

Each component was scored on a scale from 0 (lowest performance) to 4 (highest performance). The overall score, obtained by summing the three subscores, ranged from 0 to 12. A total score of ≤8 was considered indicative of reduced physical performance (8).

#### 2.3.3 Body composition assessment

##### 2.3.3.1 Anthropometric measurements

Patients self-reported their habitual body weight (kg) corresponding to the previous 6–12 months. Current body weight (kg) was measured using a calibrated scale (SECA, Birmingham, UK), and height (m) was determined with a stadiometer from the same manufacturer. Weight loss was calculated as: (habitualweight–currentweight)/habitualweight×100 , and body mass index (BMI) was derived as $\text{weight}/\text{heigh}t^{2}(\mathrm{kg}/m^{2})$ .

Calf circumference (CC) was measured at the point of greatest calf girth using a flexible measuring tape, expressed in centimeters. The appendicular skeletal muscle mass index (ASMI) was estimated using a validated predictive equation incorporating CC (cm), age (years), height (m), and sex (25).

##### 2.3.3.2 Bioimpedance vector analysis (BIVA)

Phase angle and body composition parameters were assessed using a phase-sensitive bioelectrical impedance analyzer operating at 50 kHz (Nutrilab®, Akern, Florence, Italy). The phase angle (PA) was calculated in degrees as the arctangent of reactance to resistance (Xc/R) × (180°/*π*). A standardized phase angle (SPA) was derived by adjusting PA values according to sex and age.

Body composition variables obtained from bioelectrical impedance vector analysis (BIVA) included fat-free mass (FFM/height, kg/m), fat mass (FM/height, kg/m), total body water (TBW/height, kg/m), extracellular water (ECW as a percentage of TBW), body cell mass (BCM/height, kg/m), skeletal muscle mass index (SMI, kg/m^2^), and appendicular skeletal muscle mass (ASMM, kg), which were estimated using validated predictive equations (26, 27). Reference values provided by the Nutrilab® software were applied (28).

BIVA (BIVA) was performed following standardized procedures to ensure both reliability and reproducibility. Participants were positioned supine with limbs slightly separated to avoid skin contact, which could affect impedance readings and to maintain postural stability. Prior to data collection, a five-minute rest period in the supine position was observed to minimize fluid redistribution associated with posture changes. The BIVA instrument was calibrated daily in accordance with the manufacturer’s instructions, and measurement accuracy was confirmed using a precision verification circuit provided by the manufacturer.

##### 2.3.3.3 Nutritional ultrasound

Muscle ultrasonography was carried out on the quadriceps rectus femoris (QRF) of the lower limb using a 10–12 MHz multifrequency linear probe (Mindray Z60, Madrid, Spain). All participants were examined in a supine position, and care was taken to avoid applying compression during measurements. The probe was placed at the lower third of the distance between the anterior superior iliac spine and the superior pole of the patella. The assessment included measurements of anteroposterior muscle thickness, circumference, and cross-sectional area. A single operator, trained in musculoskeletal ultrasonography, conducted all scans, ensuring the probe was perpendicular to both the longitudinal and transverse axes of the QRF. Measured parameters included rectus femoris cross-sectional area (RF-CSA), rectus femoris circumference (RF-CIR), RF axes (*X*-axis and *Y*-axis), and leg subcutaneous adipose tissue (L-SAT). Each parameter was measured in triplicate, and the mean value was used for analysis.

For abdominal adipose tissue evaluation, the midpoint between the xiphoid process and the navel was identified for imaging. Measurements included total subcutaneous abdominal fat (T-SAT), superficial subcutaneous abdominal fat (S-SAT), and visceral or preperitoneal fat (VAT), all expressed in centimeters (29).

### 2.4 Statistical analysis

Statistical analyses were conducted using IBM SPSS version 25.0 (IBM, New York, NY, USA), and graphical outputs were generated with R software v.3.5.1 (RStudio, PBC, Boston, MA, USA). The normality of quantitative variables was assessed using the Shapiro–Wilk test. Continuous variables are presented as mean ± standard deviation, and comparisons between paired observations according to sarcopenia status (presence or absence) were performed using Student’s *t*-test or, when normality assumptions were not met, the Wilcoxon signed-rank test. Categorical variables are expressed as proportions, and differences between groups were evaluated using the Chi-square test, with Fisher’s exact test applied when appropriate. Pearson correlation coefficients were calculated to examine relationships between continuous variables.

Logistic regression models were constructed to explore the associations between sarcopenia, NIMV use, and morphofunctional measurements. Independent variables included those showing significant differences in *t*-tests (*p* < 0.05) between sarcopenic and non-sarcopenic participants, as well as between NIMV users and non-users, along with clinically relevant variables. Models were adjusted for sex and age to account for potential physiological influences on the outcomes of interest.

The predictive performance of morphofunctional and respiratory variables was evaluated using receiver operating characteristic (ROC) curves and the area under the curve (AUC). A *p*-value < 0.05 was considered statistically significant.

## 3 Results

### 3.1 Comparison of study population characteristics based on sarcopenia status

The study evaluated various demographic, clinical, body composition, respiratory and functional data in 40 ALS patients.

Table 1 presents the characteristics of the study population stratified by sarcopenia status. The two groups were similar in terms of age, sex, disease duration, and type of disease onset. As anticipated, malnutrition was markedly more common among participants with sarcopenia compared to those without (90% vs. 25%; *p* < 0.001). No significant differences were observed in the type of disease onset, bulbar versus spinal, between the groups (53% vs. 56%; *p* = 0.71).

**Table 1:** Baseline characteristics and comparisons between patients with and without sarcopenia.

	Total (*n* = 40)	Non-sarcopenia (*n* = 30, 75%)	Sarcopenia (*n* = 10, 25%)	*p*-value
Demographic data				
Gender [%(*n*)]				0.56
Men	67 (27)	70 (21)	60 (6)	
Woman	33 (13)	30 (9)	40 (4)	
Age (years)	66 ± 10.5	65.1 ± 11.4	68.8 ± 6.7	0.34
Clinical data				
Disease evolution time (months)	26.6 ± 25.4	22.6 ± 16.3	19.9 ± 12.7	0.65
Onset [%(*n*)]				0.71
Bulbar	55 (22)	53 (16)	60 (6)	
Spinal	45 (18)	46 (14)	40 (4)	
Malnutrition [%(*n*)]	42 (16)	25 (7)	90 (9)	<0.001
Anthropometric measures				
BMI (kg/m^2^)	24.4 ± 4.9	25.8 ± 4.3	20.3 ± 4.2	<0.001
CC (cm)	34.6 ± 4.1	35.9 ± 3.3	30.2 ± 3	0.01
ASMI (kg/m^2^)	6.8 ± 1.6	7.2 ± 1.4	5.6 ± 1.5	0.01
BIVA				
PA (°)	4.7 ± 0.8	4.9 ± 0.8	4.1 ± 0.8	0.01
SPA	−0.6 ± 1.2	−0.5 ± 1.1	−0.7 ± 1.7	0.70
TBW (%)	56 ± 7.2	55.4 ± 7.4	58 ± 6.5	0.35
ECW (%/TBW)	53.1 ± 5.3	51.8 ± 4.6	57.1 ± 5.7	0.01
FFMI (kg/m^2^)	17.9 ± 2.6	18.7 ± 2.1	15.1 ± 1.6	<0.001
FMI (kg/m^2^)	6.3 ± 3.6	6.8 ± 3.7	4.3 ± 2.7	0.06
BCMI (kg/m^2^)	8.3 ± 2	8.9 ± 1.7	6.3 ± 1.3	<0.001
SMI (kg^2^/m^2^)	8.3 ± 1.7	8.9 ± 1.5	6.6 ± 1.3	<0.001
Nutritional ultrasound				
RF-Y axis (cm)	1.2 ± 0.4	1.3 ± 0.4	1.0 ± 0.3	0.15
RF-CSA (cm^2^)	3.6 ± 1.6	3.8 ± 1.7	3.1 ± 0.9	0.22
L-SAT (cm)	0.7 ± 0.6	0.8 ± 0.7	0.6 ± 0.3	0.34
T-SAT (cm)	1.5 ± 0.9	1.5 ± 0.9	1.4 ± 0.7	0.70
VAT (cm)	0.6 ± 0.4	0.7 ± 0.4	0.4 ± 0.2	0.10
Functional status				
HGS max (kg)	20.5 ± 11.1	22.9 ± 11.4	13.3 ± 6.3	0.02
SPPB	7.1 ± 3.1	7.7 ± 2.6	5.5 ± 4	0.06
ALSFRS-R	35.5 ± 8.8	37.9 ± 7.5	28.7 ± 9.1	0.01
Respiratory assessment				
FVC (%)	74.8 ± 24.2	79.5 ± 22.3	53.6 ± 22.0	0.02
PCF (L)	286 ± 148	307 ± 152	215 ± 112	0.12
pCO_2_ (mmHg)	43 ± 8.1	41.2 ± 7.7	48.3 ± 7.1	0.02
NIMV [%(*n*)]	25.6 (10)	10.3 (3)	70.0 (7)	<0.001
CA [%(*n*)]	23 (9)	10 (3)	60 (6)	0.01

Results are expressed as mean ± SD (numeric variables) or %(*n*) (categorical variables). ALSFRS-r: revised Amyotrophic lateral sclerosis functional rating scale (0–48); ASMI: appendicular skeletal mass index; BMI, body mass index; BCMI, body cell mass index; BIVA, bioimpedance vectorial analysis; CA, cough assist; CC, calc circumference; ECW, extracellular water; FVC, forced vital capacity; FFMI, fat free mass index; FMI, fat mass index; HGS, hand grip strength; NIMV, non-invasive mechanical ventilation; PA, phase angle; PCF, peak cough flow; pCO_2_, CO_2_ pressure; SPPB, short physical performance battery; RF-CSA, rectus femoris cross-sectional area; SAT, abdominal subcutaneous adipose tissue abdominal (T) and leg (L); SMI, skeletal muscle index; SPA, standardized phase angle; VAT, preperitoneal or visceral adipose tissue.

Muscle status was characterised with a prevalence of 53% of dynapenia, 46% myopenia and 67% frailty, resulting in 25% sarcopenia prevalence in our sample.

Related to anthropometric, BIVA-body composition, nutritional ultrasound and functional status assessment of the study population, sarcopenia patients showed significantly lower BMI (25.8 ± 4.3 kg/m^2^ vs. 20.3 ± 4.2 kg/m^2^; *p* < 0.001), CC value (35.9 ± 3.3 cm vs. 30.2 ± 3 cm; *p* = 0.001) and ASMI (7.2 ± 1.4 kg/m^2^ vs. 5.64 ± 1.5 kg/m^2^; *p* = 0.003). Similarly, BIVA variables related to muscle mass were lower in the sarcopenia group: FFMI (18.7 ± 2.1 vs. 15.1 ± 1.6 kg/m^2^; *p* < 0.001), BCMI (8.9 ± 1.7 vs. 6.3 ± 1.3 kg/m^2^; *p* < 0.001) and SMI (8.9 ± 1.5 vs. 6.6 ± 1.3 kg^2^/m^2^; *p* < 0.001). However, there were no differences between variables measured by ultrasound neither adipose tissue obtained by BIVA. Additionally, ALS patients with sarcopenia showed significantly decreased PA (4.9 ± 0.8 vs. 4.1 ± 0.8; *p* = 0.009) and increased ECW (51.8 ± 4.6 vs. 57.1 ± 5.7; *p* = 0.008). Besides, sarcopenia group had significantly less strength (22.9 ± 11.4kg vs. 13.3 ± 6.3; *p* = 0.02) and more severe degree of disease according to ALSFRS-R (37.9 ± 7.5 vs. 28.7 ± 9.1; *p* = 0.003). However, SPPB did not show significant differences.

In terms of respiratory variables, sarcopenia patients presented worse respiratory: lower FVC (53.6% ± 22.0 vs. 79.5% ± 22.3; *p* = 0.02), higher pCO_2_ (48.3 mmHg ± 7.1 vs. 41.2 mmHg ± 7.7; *p* = 0.02), higher prevalence of VMNI (70% vs. 10%, *p* = 0.001) and CA use (60% vs. 10%, *p* = 0.001).

### 3.2 Correlation between disease severity, morphofunctional and respiratory parameters

**Figure 1:**
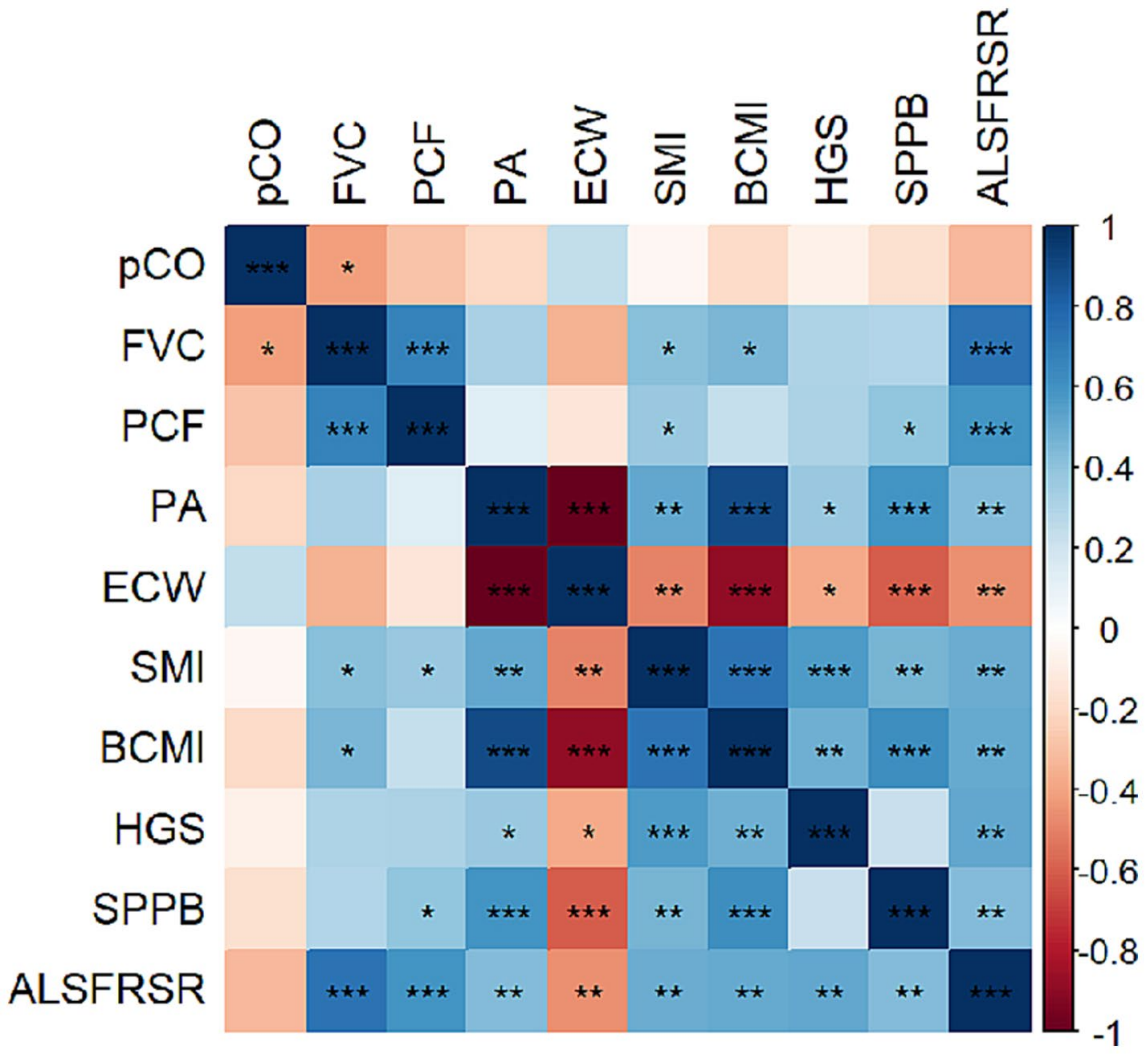
Pearson’s correlation plot of morphofunctional and disease evolution scores. Red colors indicate negative correlation, while blue colors positive correlations. Color intensity represents the strength of the correlation, with deeper shades signifying stronger relationships. Asterisks (*) indicate significant correlation between variables according to the Pearson’s correlation test (*p < 0.05; **p < 0.01; ***p < 0.001). ALSFRSR, revised Amyotrophic lateral sclerosis functional rating scale; BCMI, body cell mass index; FVC, forced vital capacity; HGS, hand grip strength; PA, phase angle; PCF, peak cough flow; pCO, CO_2_ pressure; SPPB, short physical performance battery.

Significant positive correlations were found with BIVA muscle mass variables BCMI (FVC: *r* = 0.450, *p* = 0.01), SMI (FVC: *r* = 0.413, *p* = 0.02; PCF: *r* = 0.371, *p* = 0.03) and ALSFRS-R (FVC: *r* = 0.731, *p* < 0.001; PCF: *r* = 0.592, *p* < 0.001). ALSFRS-R were associated with all morphofunctional and respiratory parameters. All results are detailed in Figure 1.

### 3.3 Morphofunctional, respiratory and clinical predictors of sarcopenia and NIMV: risk associations

**Figure 2:**
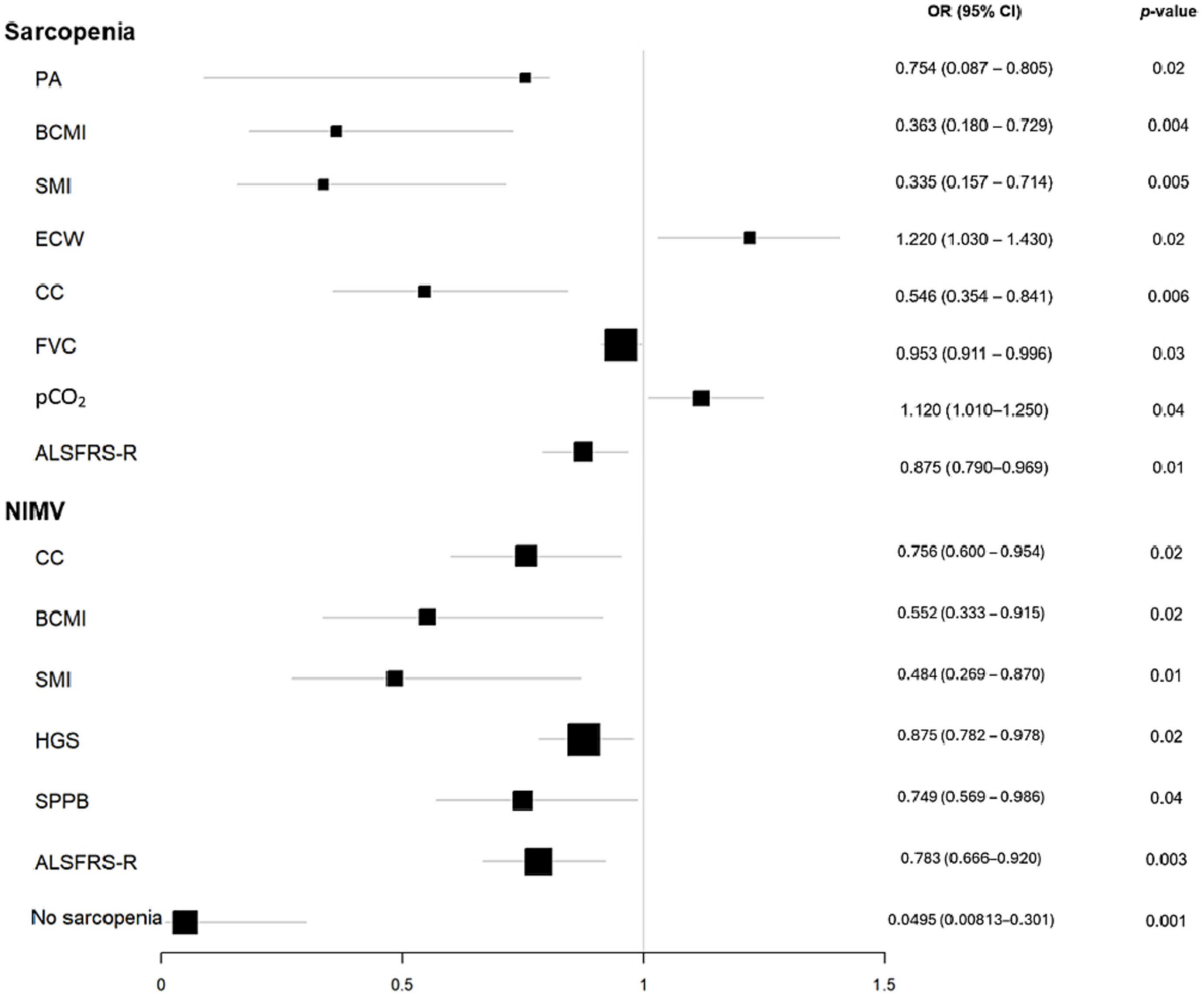
Sarcopenia and NIMV odds ratio plot. The size of the black squares indicates the weight that the result of each variable has on the final result (sarcopenia or NIMV). The bigger size, the lower variability. ALSFRSR, revised Amyotrophic lateral sclerosis functional rating scale; BCMI, body cell mass index; CC, calf circumference; FVC, forced vital capacity; HGS, hand grip strength; PA, phase angle; PCF, peak cough flow; pCO, CO_2_ pressure; SPPB, short physical performance battery.

Morphofunctional variables were significantly associated with sarcopenia risk. Specifically, each 1-point increase in PA corresponded to a 73.5% reduction in risk, while higher BCMI was linked to a 63.7% lower risk. Conversely, a higher %ECW was associated with a 22% increase in sarcopenia risk (Figure 2). No significant associations were observed for nutritional ultrasound parameters. Malnutrition was strongly related to sarcopenia, with an OR of 27.0 (95% CI: 2.9–252.6, *p* = 0.004).

Regarding respiratory and functional variables, each unit increase in FVC was associated with a 5% reduction in sarcopenia risk, whereas higher pCO₂ levels increased the risk by 12%. Additionally, higher ALSFRS-R scores were linked to a 12.5% decrease in sarcopenia risk. Notably, patients without sarcopenia had a 95% lower likelihood of requiring NIMV.

Morphofunctional variables were associated with the use of NIMV. Specifically, an increase in CC, SPPB, BCMI, SMI, HGS and ALSFRS-R was associated with a 24.4, 25.1, 44.8, 51.6, 10.4 and 12.5% decreased risk, respectively. No associations were found in nutritional ultrasound variables (Figure 2).

### 3.4 Morphofunctional and respiratory variables predictors of sarcopenia: cut-off values

The identified cut-off values for predicting sarcopenia were as follows: PA 4.65°, BCMI 8.05 kg/m^2^, ECW 53%, FVC 54.2%, pCO_2_ 43.5 mmHg and ASLFRS-R 28 points (Table 2). Among these, BCMI demonstrated the most favorable predictive performance, with a cut-off of 8.05 kg/m^2^, yielding an AUC of 0.889, sensitivity of 88.9%, and specificity of 71.4%. In addition, pCO_2_ showed a high sensivity (88.9%) and FVC a high specificity (88.9%).

**Table 2:** Optimal cut-off points and diagnostic performance of variables for sarcopenia detection.

Variable	Cut-off point	Sensitivity (%)	Specificity (%)	False positive rate (%)	False negative rate (%)	AUC
PA (°)	4.65	77.8	75.0	25.0	22.2	0.754
BCMI (kg/m^2^)	8.05	88.9	71.4	28.6	11.1	0.889
ECW (%)	53	77.8	78.6	21.4	22.2	0.758
FVC (%)	54.2	66.7	88.9	11.1	33.3	0.793
pCO2 (mmHg)	43.5	88.9	77.8	22.2	11.1	0.790
ALSFRS-R	27	50.0	96.4	3.6	50.0	0.777

PA, phase angle; BCMI, body cell mass index; ECW, extracellular water; FVC, forced vital capacity; pCO_2_, CO_2_ pressure; ALSFRS-R, amyotrophic lateral sclerosis functional rating scale-revised.

### 3.5 Morphofunctional and clinical variables predictors of non-invasive mechanical ventilation need: cut-off values

The cut-off values identified for predicting the need for NIMV was CC 31.75 cm, BCMI 8.15 kg/m^2^, SMI 8.25 kg/m^2^, HGS 13 kg, SPPB 7 points and ASLFRS-R 33 points (Table 3). ALSFRS-R emerged as the most reliable predictor, with a threshold of 33 points, demonstrating an AUC of 0.884, correctly identifying 80% of participants who required NIMV, and correctly excluding 75% of those who did not. BCMI showed strong ability to identify true positives, with a sensitivity of 88.9%, whereas HGS provided the highest ability to correctly identify true negatives, with a specificity of 84.6%.

**Table 3:** Performance metrics for different variables with specified cut-off points in NIMV.

Variable	Cut-off point	Sensitivity (%)	Specificity (%)	False positive rate (%)	False negative rate (%)	AUC
CC (cm)	31.75	70	82.1	17.5	30	0.755
BCMI (kg/m^2^)	8.15	88.9	63	37	11.1	0.776
SMI (kg/m^2^)	8.25	77.8	63	37	22.2	0.788
SPPB	7	60	65.5	34.5	40	0.664
HGS (kg)	13	66.7	84.6	15.4	33.3	0.782
ALSFRS-R	33	80	75	25	20	0.884

CC, calc circumference; BCMI, body cell mass index; SMI, skeletal muscle index; SPPB, short physical performance battery; HGS, handgrip strength; ALSFRS-R, amyotrophic lateral sclerosis functional rating scale-revised.

## 4 Discussion

This study investigated the prevalence of sarcopenia in patients with ALS and analyzed its impact on respiratory function and disease severity in ALS patients. Our findings demonstrated that sarcopenia was present in one out of four ALS patients. We confirmed the presence of poorer nutritional status, reduced respiratory capacity, and greater functional impairment and disease progression in ALS sarcopenic group. Moreover, we identified key predictors of sarcopenia and the need of NIMV. Specifically, lower values of PA, BCMI, FVC, CC and ALSFRS-R, along with higher %ECW and pCO₂, were linked to a greater risk of sarcopenia. In parallel, decreased CC, BCMI, SMI, HGS, SPPB, and ALSFRS-R values were associated with a higher risk of requiring ventilatory support.

To the best of our knowledge, no studies have specifically investigated the prevalence of sarcopenia in patients with ALS. However, research in other neuromuscular diseases, such as multiple sclerosis, has reported a sarcopenia prevalence similar prevalence rates, around 20% (30). While interest in sarcopenia in ALS is increasing, it remains largely understudied compared to other clinical factors such as malnutrition (9). Recently, Azzolino et al. (6) proposed that ALS may serve as an accelerated model of sarcopenia, due to shared characteristics such as muscle atrophy, systemic inflammation, malnutrition, and reduced mobility. This perspective underscores the importance of assessing sarcopenia as an intrinsic component of ALS progression with potential prognostic and therapeutic implications (13).

The similar prevalence of bulbar-onset disease between sarcopenic and non-sarcopenic patients suggests that reduced oral intake alone does not fully explain muscle loss in ALS. Beyond dysphagia-related malnutrition, hypermetabolism has been increasingly recognized as a key contributor to weight loss, muscle wasting, and adverse prognosis in ALS (31). Elevated resting energy expenditure and altered substrate utilization may accelerate catabolic processes, loss of motor neurons, thereby promoting sarcopenia even in the absence of severe feeding impairment (6, 32).

In the assessment of nutritional status, our findings revealed a substantial overlap between malnutrition and sarcopenia, with a higher proportion of malnourished patients among those diagnosed with sarcopenia. Previous studies have reported similar body composition alterations among malnourished ALS patients (33, 34). In line with these findings, we observed significant differences in anthropometric measurements, BIVA, and functional parameters according to sarcopenia status. These results highlighted the strong association between poor nutritional status, muscle mass loss, and functional decline in ALS.

We observed lower PA and muscle mass indices (ASMI, FFMI, BCMI, SMI) in the sarcopenic group, consistent with previous research showing that sarcopenic older adults had reduced PA and lean body mass (35). Moreover, sarcopenic ALS patients demonstrated higher %ECW despite no significant differences in TBW. Interestingly, %ECW was inversely associated with muscle function (SPPB), strength (HGS), and muscle mass markers (SMI, BCMI, ASMI). Previous studies highlighted that changes in body fluid distribution, assessed by BIA could serve as a indicators of muscle wasting and strength decline (36). In critically ill patients, these hydration shifts were linked to increased proteolysis and reduced protein synthesis, suggesting that extracellular fluid expansion might reflect muscle atrophy rather than true alterations in TBW (37).

In our cohort, PA, BCMI, SMI and %ECW were all associated with sarcopenia risk. These observations aligned with previous evidence identifying PA as a biomarker of cellular integrity and a predictor of sarcopenia in various clinical populations, including chronic musculoskeletal diseases and pulmonary conditions (38). Notably, BCMI emerged as the most discriminative biomarker of sarcopenia in our study, with a threshold that not only identified individuals at risk of sarcopenia but also aligned closely with the need for non-invasive mechanical ventilation. This reinforces its clinical utility as a sensitive indicator of muscle mass depletion and nutritional deterioration. Previous studies have also supported the value of BCMI as a reliable marker for muscle status and disease severity in neuromuscular conditions (39), underscoring its potential role in early risk stratification and therapeutic decision-making in ALS. The PA cut-off identified here also echoed previous reports highlighting its prognostic value in ALS (17).

On the other hand, frailty was highly prevalent in our cohort. SPPB scores were closely associated with strength (HGS), muscle mass (BCMI, SMI), FVC, and overall functional status (ALSFRS-R), underscoring the importance of assessing physical performance in ALS patients. Although SPPB scores did not differ significantly between sarcopenic and non-sarcopenic patients, we observed a clear trend toward lower performance in the sarcopenic group. This trend suggested a potentially meaningful clinical association that may have been underestimated due to the limited sample size. Indeed, prior studies in older adults demonstrated that SPPB scores ≤8 were associated with increased risk of sarcopenia, frailty, and mortality (40). Furthermore, emerging evidence proposed frailty not only as a predictor of adverse outcomes but also as a potential risk factor for ALS development (41).

Muscle strength plays a crucial role in ALS progression. HGS was widely used to assess muscle strength and is valued for its sensitivity to rapid decline over time, making it a useful marker for or monitoring disease progression (29, 30). In our cohort, ALS disease severity, measured by ALSFRS-R, showed a positive correlation with HGS, consistent with the findings of Shefner et al. (42). Moreover, we identified ALSFRS-R cut-off values for sarcopenia diagnosis and the need of NIMV. This aligned with reported evidence suggesting that functional decline captured by ALSFRS-R is closely linked to both nutritional deterioration and respiratory impairment (43). Additionally, the concept of “respiratory sarcopenia” proposed by Nagano et al. (13) described a bidirectional relationship between respiratory dysfunction and skeletal muscle decline. In their study, patients with low FVC and reduced maximal inspiratory pressure showed significantly decreased appendicular lean mass and handgrip strength. These findings supported the idea that generalized muscle atrophy, including respiratory muscles, may accelerate ventilatory decline and overall disease progression in ALS.

Furthermore, respiratory function is a major determinant in ALS progression and is closely linked to skeletal muscle strength and mass (42, 44). Large longitudinal studies have shown that respiratory dysfunction follows a progressive restrictive pattern, with declines in FVC, slow vital capacity (SVC), SNIP, and PCF, and that ALSFRS-R respiratory subscores correlate only modestly with objective respiratory measures (45). In our study, sarcopenic patients had significantly lower FVC values, higher pCO₂ and a greater need for NIMV use. Respiratory parameters (FVC, PCF) were correlated positively with muscle status (BCMI, SMI, PA) and ALSFRS-R scores. This is aligned with the role of generalized muscle loss in respiratory impairment in ALS (42, 44, 46, 47).

Although direct measurements of respiratory muscle strength were not available, previous studies have demonstrated that diaphragmatic dysfunction occurs early in ALS and contributes substantially to ventilatory impairment, even when upright FVC remains preserved (48, 49). In this context, our findings suggest that generalized muscle loss, reflected by reduced muscle mass and quality, may parallel or exacerbate respiratory muscle impairment. Nevertheless, limb muscle weakness and respiratory muscle involvement may not progress synchronously in all patients, and peripheral sarcopenia may occur independently of diaphragmatic weakness in some cases (50).

In line with this, we observed higher FVC values was associated with a lower risk of sarcopenia, whereas elevated pCO₂ levels were linked to an increased risk. In line with these findings, Iglesias et al. demonstrated that patients with lower diaphragmatic excursion, measured by ultrasound, were associated with decreased FVC and increased respiratory burden (51). Declines in FVC have been consistently identified as strong predictors of respiratory insufficiency, need for ventilatory support, and mortality in ALS (52). Accordingly, current clinical guidelines recommend the use of FVC, respiratory muscle strength assessment, and nocturnal monitoring to guide timely initiation of non-invasive ventilation, given their established prognostic value (53).

In our cohort, patients without sarcopenia were less likely to need NIMV. Specifically, muscle mass (BCMI, SMI), strength (HGS), physical performance (SPPB) and global disease status (ALSFRS-R) were all associated with lower risk of needing ventilatory support. These findings reinforced the hypothesis that progressive muscle loss not only compromises mobility and independence but also directly impacts respiratory function. Similar patterns were observed in other neuromuscular and lung diseases, where muscle depletion correlated with poorer respiratory outcomes (54). While functional and respiratory measures such as FVC and ALSFRS-R were established prognostic indicators of respiratory dysfunction in ALS, few studies have specifically examined the contribution of muscle status to ventilatory dependence (55). Our data suggested that integrating muscular and nutritional assessments into routine evaluation may improve early identification of patients at heightened risk for respiratory decline.

Typically, malnutrition was identified as a prognostic factor for sarcopenia, due to its impact on muscle catabolism and metabolic imbalance (56). In ALS, malnutrition and reduced skeletal muscle mass were associated with elevated plasma neurofilament light chain levels and faster disease progression. These findings suggest that muscle loss in ALS is not merely a consequence of denervation and disuse but may also reflect systemic disease activity and neurodegenerative burden (57). Similarly, our analysis revealed that malnutrition was the strongest predictor of sarcopenia. These results highlighted the overlap and interdependence between nutritional deterioration, muscle status and respiratory vulnerability in ALS.

The current study has several notable strengths. To our knowledge, it is the first to report the prevalence of sarcopenia in ALS patients, providing novel insights into its clinical relevance. We applied standardized diagnostic criteria from leading scientific societies: GLIM for malnutrition and EWGSOP2 for sarcopenia. This improves the comparability and reproducibility of our results and facilitates future cross-study analyses. A key strength is comprehensive morphofunctional assessment. We evaluated muscle mass, strength, and function, covering all relevant dimensions. In addition, this work provides an initial framework to identify the most useful morphofunctional tools for clinical practice. It helps to distinguish those most effective in detecting sarcopenia risk and ventilatory impairment. Finally, we demonstrated a correlation between body composition, muscle function, and respiratory status. This reinforces the connection between nutritional, muscular, and respiratory decline in ALS.

In contrast, this study has several limitations. The small sample size reduces the statistical power and limits the development of robust predictive models for sarcopenia. Additionally, the cross-sectional design prevents the establishment of causal relationships. It limits our ability to assess the progression of sarcopenia and respiratory decline over time. The sample was heterogeneous in disease duration, which is an inherent characteristic of ALS but may have influenced the variability in results. Direct respiratory muscle strength measurements (MIP, MEP, SNIP) and comprehensive sleep evaluations were not available for all patients. Therefore, respiratory analyses were based on routinely collected parameters (FVC%, PCF, and PaCO₂). Patients with known severe chronic respiratory diseases were excluded, but 5% were found to have mild chronic obstructive pulmonary disease. We did not adjust for co-existing respiratory diseases in the analysis, which may have independently influenced FVC measurements and the assessment of respiratory decline. Although we used the ALSFRS-R as a measure of functional impairment, which is closely associated with quality of life in ALS, we acknowledge that dedicated quality of life instruments could provide complementary insights. This represents a limitation of the present study and an important area for future research. Furthermore, the inclusion of patients at different disease stages adds complexity to data interpretation. Finally, this was a single-center study. As such, the findings may reflect local clinical practices and may not be fully generalizable to broader ALS populations.

## 5 Conclusion

In summary, this study provides the first report on the prevalence of sarcopenia in patients with ALS, revealing its close association with nutritional deterioration, functional decline, and respiratory compromise. Using standardized diagnostic criteria and a comprehensive morphofunctional assessment, we identified key parameters — including BCMI, SMI, PA, %ECW, HGS, SPPB, FVC, and ALSFRS-R — that were significantly associated with sarcopenia risk and ventilatory dependence. These findings emphasize the clinical importance of early identification and monitoring of muscle mass, strength, and function, as muscle depletion impacts not only mobility but also respiratory capacity and overall disease progression. Incorporating muscular and nutritional assessments into routine ALS management may enhance risk stratification and inform tailored interventions. Future longitudinal studies are needed to validate these associations over time and to explore strategies that may mitigate the impact of sarcopenia on ALS outcomes.

## Transparency statement

All authors confirm that the present manuscript is a transparent and honest account of the reported research. This study is related to our previous publication (*Zarco-Martín et al.*, *Nutrients* 2024;16:2625), which focused on malnutrition in ALS. The current study explores sarcopenia and its respiratory implications as a complementary analysis within the same research line. Both studies were conducted within the ALS Multidisciplinary Unit (UMELA) at San Cecilio University Hospital and are part of the same research project approved by the Ethics Committee of Granada (ID: 1770-N-21). A partial overlap exists between cohorts, as both derive from the same prospective registry. However, the present analysis includes additional patients and focuses on different diagnostic constructs (sarcopenia vs. malnutrition) and outcome measures (respiratory and functional parameters), ensuring the originality and independence of this work.

## Data Availability

The raw data supporting the conclusions of this article will be made available by the authors, without undue reservation.
